# Validation of the Attitudes Towards Psychological Online Interventions Questionnaire Among Black Americans: Cross-cultural Confirmatory Factor Analysis

**DOI:** 10.2196/43929

**Published:** 2023-04-27

**Authors:** Donovan Michael Ellis, Page Lyn Anderson

**Affiliations:** 1 Department of Psychology Georgia State University Atlanta, GA United States

**Keywords:** acceptability, Black American, iCBT, internet-based cognitive behavioral therapy, digital treatment, confirmatory factor analysis, bifactor model

## Abstract

**Background:**

Acceptability of digital mental health interventions is a significant predictor of treatment-seeking behavior and engagement. However, acceptability has been conceptualized and operationalized in various ways, which decreases measurement precision and leads to heterogeneous conclusions about *acceptability*. Standardized self-report measures of acceptability have been developed, which have the potential to ameliorate these problems, but none have demonstrated evidence for validation among Black communities, which limits our understanding of attitudes toward these interventions among racially minoritized groups with well-documented barriers to mental health treatment.

**Objective:**

This study aims to examine the psychometric validity and reliability of one of the first and most widely used measures of acceptability, the Attitudes Towards Psychological Online Interventions Questionnaire, among a Black American sample.

**Methods:**

Participants (N=254) were recruited from a large southeastern university and the surrounding metropolitan area and completed the self-report measure via a web-based survey. A confirmatory factor analysis using mean and variance adjusted weighted least squares estimation was conducted to examine the validity of the underlying hierarchical 4-factor structure proposed by the original authors of the scale. An alternative, hierarchical 2-factor structure model and bifactor model were examined for comparative fit.

**Results:**

The findings indicated that the bifactor model demonstrated a superior fit (comparative fit index=0.96, Tucker-Lewis index=0.94, standardized root mean squared residual=0.03, and root mean square error of approximation=0.09) compared with both 2- and 4-factor hierarchical structure models.

**Conclusions:**

The findings suggest that, within a Black American sample, there may be greater utility in interpreting the Attitudes Towards Psychological Online Interventions Questionnaire subscales as attitudinal constructs that are distinct from the global *acceptability* factor. The theoretical and practical implications for culturally responsive measurements were explored.

## Introduction

### Background

Black communities face persistent barriers to mental health treatment, including cost, accessibility, and stigma [1-3]. Internet-based psychological interventions that implement evidence-based techniques, including psychoeducation, behavioral activation, mindfulness strategies, and symptom tracking [4], may prove useful for improving equitable access to mental health treatment as they are often more cost-effective [5,6], private [7], and readily accessible [8]. Digital interventions that are empirically driven and incorporate elements of cognitive behavioral therapy are typically referred to as internet-based cognitive behavioral therapy (iCBT) [9]. People benefit from iCBT when paired with therapist support or used alone, although the magnitude of the effect is often higher for programs with therapist assistance [10,11] (for more conservative findings on the comparative benefit of therapist support with iCBT, see the study by Bernstein et al [12]). Although iCBT programs are effective for a variety of anxiety, mood, and substance use disorders [13,14], studies have consistently reported their underutilization by the public [15,16].

### Acceptability of iCBT

Studies examining this research-to-practice gap have revealed a complex picture of user acceptance of digital mental health interventions. Although therapist-supported iCBT is generally rated as more acceptable than self-guided programs [17,18], the overall willingness to use iCBT is low. In one study, 16% of non–treatment-seeking adults reported a willingness to consider using a digital mental health intervention to address a mental health concern [19], and another study reported that only 12% of participants were “definitely interested” in internet-based treatment [20]. Overall, people reported that they significantly preferred face-to-face therapy over iCBT and other digital mental health interventions [20,21].

A problem in this budding literature is that the construct of acceptability has been defined in a variety of ways, which may contribute to heterogeneous results regarding consumer attitudes toward iCBT [22]. Retrospective study outcomes, such as treatment satisfaction, engagement, usability, and feasibility, are often used interchangeably with acceptability [23]. Other researchers propose more prospective metrics, conceptualizing acceptability as “cognitively based, positive attitudes towards such interventions” that aim to predict treatment seeking [24]. Acceptability has sometimes been operationalized with measures of similar constructs, such as outcome expectancy—the expectation that one will benefit from treatment [25]. In some studies, acceptability was operationalized using single Likert scale items measuring willingness to use an intervention [20,26,27], and in other studies, researchers developed their own measure of acceptability [19,28]. The lack of precision in conceptualization and measurement may explain why conclusions about the acceptability of iCBT vary widely across studies.

A total of 6 self-report measures of consumer acceptability of digital mental health interventions now exist, with evidence of their psychometric properties and factor structure [24,29-33]. However, reflecting existing heterogeneity in the literature, these measures operationalize acceptability in various ways. The Attitudes Towards Psychological Online Interventions (APOI) questionnaire conceptualizes acceptability as a set of positive and negative appraisals and is designed to be used with various forms of digital mental health interventions [24]. The e-Therapy Attitudes and Process Questionnaire [29] includes items specifically related to users’ anticipated engagement with and short-term adherence to digital interventions. The Online Psychoeducational Intervention–Brief Attitudes Scale [32] is an abbreviated measure of attitudes (5 items) that makes the conceptual distinction that attitudes toward web-based psychoeducational interventions should incorporate elements of both psychotherapy and learning methods. In addition, 3 measures have been developed to assess working alliances in different digital contexts, akin to the therapeutic alliance fostered in face-to-face therapy [34]. The Working Alliance Inventory for guided internet interventions [30] measures the perception of an emotional attachment or collaborative bond with a digital mental health intervention, and the Working Alliance Inventory applied to virtual and augmented reality [33] measures participant comfort and trust in a virtual reality environment. Similarly, the Virtual Therapist Alliance Scale [31] measures perceptions of the therapeutic alliance with digital therapist avatars common to automated virtual reality exposure therapies. Table 1 shows the characteristics of the acceptability measures.

**Table 1:** Measures of acceptability toward digital mental health interventions.

Study	Title	Abbreviation	Intervention modality
Clough et al [29], 2019	e-Therapy Attitudes and Process Questionnaire	eTAP	All
Gómez Penedo et al [30], 2020	Working Alliance Inventory for Guided Internet Interventions	WAI-I	Guided interventions
Miloff et al [31], 2020	Virtual Therapist Alliance Scale	VTAS	Augmented and virtual reality
Miragall et al [33], 2015	Working Alliance Inventory Applied to Virtual and Augmented Reality	WAI-VAR	Augmented and virtual reality
Schröder et al [24], 2015	Attitudes Towards Psychological Online Interventions Questionnaire	APOI	All
Teles et al [32], 2021	Online Psychoeducational Intervention—Brief Attitudes Scale	OPI-BAS	Psychoeducation

### Racially Minoritized Communities Are Underrepresented in Acceptability Research

Further complicating matters are the dearth of acceptability research that is inclusive of ethnically or racially minoritized communities. In 1 meta-analysis, 62 of 64 randomized controlled trials examining the efficacy and acceptability of iCBT did not include (or did not report) racial minorities in their studies [13]. All but one [33] of the existing measures of consumer attitudes toward digital mental health interventions have collected data from White majority (and predominantly European language) samples [24,29-32], including the first and most highly cited measure of acceptability toward digital mental health interventions, the APOI questionnaire [24]. The APOI was developed with German-speaking participants who reported mild to moderate depression (N=1013) and were recruited from outpatient clinics, web-based health forums, and health insurance referrals.

No research to date has evaluated the reliability or validity of the APOI scale among racially or ethnically minoritized communities, including Black Americans. This is highly problematic because even though Black communities may disproportionately benefit from the advantages afforded by iCBT and related digital mental health interventions, it is unknown whether the APOI demonstrates good psychometric properties in this population.

### This Study

This study addresses this problem by assessing the psychometric properties of the APOI questionnaire in a sample of Black Americans. Using confirmatory factor analyses, this study examined whether the APOI demonstrates reliability and construct validity within a Black population. In this study, 2 measurement models were examined using 16 ordered categorical (ordinal) response items retained in the exploratory factor analysis of the APOI. The first model presents a 2-factor, hierarchical measurement model (positive and negative subfactors) distinct from the 4-factor hierarchical model proposed by Schröder et al [24]. Given considerations for equivalent models [35,36] modification indexes will be reviewed to examine new and replicative factor structures to illuminate the underlying construct of *acceptability*.

## Methods

### Recruitment

Participants were self-identified Black or African American adults (N=254 participants). The participants ranged in age from 18 to 85 (mean 27.11, SD 13.40) years and were predominantly women (172.7/254, 68%), single (167.6/254, 66%), and highly educated (at least 70% had some college education; see Table 2 for more demographic and clinical characteristics of the sample). Participants were recruited from 2 primary sources: students recruited from the participant pool of a southeastern university in an urban setting who received course credit for their participation and community participants who were solicited in public places throughout the metropolitan area (eg, parks) and had the opportunity to enter a raffle for a US $25 Amazon gift card.

**Table 2:** Demographics and clinical characteristics of participants.

Variables			Values
Age (years; n=254), mean (SD)			27.11 (13.40)
**Sex (n=254), n (%)**			
	Male	82 (32.3)	
	Female	172 (67.7)	
**Sexual identity (n=252), n (%)**			
	Heterosexual	210 (83.3)	
	Lesbian, gay, and bisexual	36 (14.3)	
	Self-identify	6 (2.4)	
**Current education status (n=253), n (%)**			
	High school	1 (0.4)	
	Some college or currently in college	173 (68.1)	
	Graduate or professional degree	5 (2.0)	
	Nondegree student or other	3 (1.2)	
	Nonstudent^a^	71 (28.0)	
**Relationship status (n=252), n (%)**			
	Single	166 (65.9)	
	Serious dating or committed relationship	55 (21.8)	
	Married or civil union	16 (6.4)	
	Separated, divorced, or widowed	15 (6.0)	
**Symptom severity,** **mean (SD)**			
	DASS^b^—total (n=243)	29.58 (20.84)	
	DASS—depression (n=250)	8.99 (8.49)	
	DASS—anxiety (n=249)	8.35 (7.10)	
	DASS—stress (n=250)	11.96 (7.88)	

^a^Reflects current noneducational status but does not indicate the highest level of education completed (ie, may include college graduates).

^b^DASS: Depression Anxiety Stress Scale.

### Procedure

Participants completed a survey developed via the Qualtrics web-based platform as part of an experimental study assessing the impact of treatment rationale on the acceptability of iCBT. Participants were randomly assigned via Qualtrics (1:1 allocation) to read either a treatment rationale or definition of iCBT (see the study by Ellis and Anderson [37] for full details). The APOI questionnaire was administered as a primary measure of acceptability. The Depression, Anxiety, and Stress Scale-21 items (DASS-21) was used to characterize the sample, as experiences of depression and anxiety have been linked to mental health treatment–seeking attitudes [38] and to provide comparative evidence to Schröder et al [24] who recruited participants with mild to moderate depression.

All the data were collected on the web and will be made available upon request.

### Measures

The APOI questionnaire [24] is a measure of attitudes toward digital mental health interventions that, for the purposes of this project, was modified to reference therapist-assisted iCBT. The development of the APOI included both exploratory and confirmatory factor analyses to identify clustering of latent constructs, resulting in 16 items comprising four subscales measuring attitudes toward psychological web-based interventions, which are as follows: (1) skepticism and perception of risk (SKE), which measures negative attitudes concerning the efficacy and security of a psychological web-based intervention; (2) confidence in effectiveness (CON), which measures positive attitudes concerning the utility and credibility of a psychological web-based intervention; (3) technologization threat (TET), which measures negative attitudes toward the lack of personal contact and the remote nature of the intervention; and (4) anonymity benefits (ABE), which measures positive attitudes related to increased privacy. Participants rate their agreement with each item (eg, “I have the feeling that iCBT can help me.”) on a 5-point Likert scale (1=totally agree to 5=totally disagree). Positively valenced items were reverse coded. The total scores ranged from 16 to 80, with higher scores indicating more positive attitudes toward iCBT. The APOI demonstrated strong overall internal consistency (Cronbach α=.77) and showed evidence of construct validity in a sample of 1013 participants [24].

The DASS-21 [39] is a measure of mental illness comprising 3 subscales: depression, anxiety, and stress. Participants rated each item on a 4-point Likert scale (0=never to 3=always). Sum scores were computed by adding the scores across items and multiplying by 2. Scores on the total DASS-21 scale ranged from 0 to 126, with higher scores indicating more distress or impairment. Scores for each subscale were determined by summing the scores for the relevant 7 items and multiplying by 2 (range 0-42). The DASS-21 demonstrates strong convergent validity with both the Beck Anxiety Inventory (*r*=0.81) and Beck Depression Inventory (*r*=0.74), indicating a satisfactory ability to discriminate between anxiety and depressive symptoms [40]. The DASS-21 was normed on a nonclinical sample (N=717), and subsequent research has supported the validity and reliability of the DASS-21 across racial groups, including Black Americans (subscales: Cronbach α=.81−.88 [41]).

### Statistical Analysis

The variables used for the factor analysis are listed in Table 3. See Tables 4 and 5 for the interitem correlation matrix and descriptive statistics.

Confirmatory factor analyses were performed using Mplus (version 8.4; Muthén & Muthén) with a sample of Black American adults (N=254) to examine the cross-cultural equivalence of the factor structure derived from the final set of 16 items indicated in the study by Schröder et al [24]. The weighted least squares means and variance adjusted (WLSMV) estimation method was used to analyze the covariance matrix structure of ordinal items. Several indices were used to evaluate the model fit: the discrepancy chi-square statistic (df≤5), standardized root mean squared residual (SRMR; SRMR≤0.08), root mean square error of approximation (RMSEA; RMSEA≤0.08), comparative fit index (CFI; CFI≥0.90), and Tucker-Lewis index (TLI; TLI≥0.90), which are commonly recommended at the indicated thresholds [42-44]. Latent variables were scaled by fixing the latent variances to 1, which allowed all indicator factor loadings to be estimated. Finally, reliability analyses of the APOI were conducted by calculating the internal consistency (Cronbach α) and corrected item-total correlations (discrimination) to facilitate comparisons with reliability metrics reported in the original publication.

**Figure 1:**
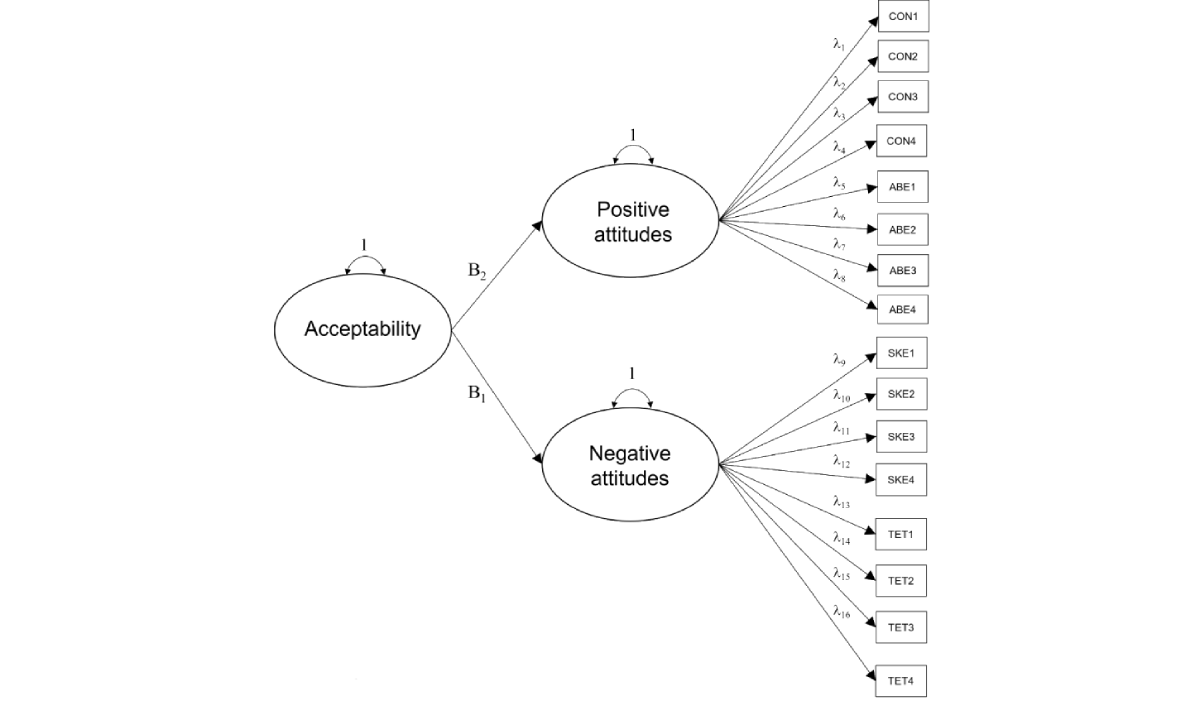
Higher-order, 2-factor model depicting hierarchical relationship among indicators of 2 latent factors: positive and negative attitudes toward treatment loading on a global acceptability factor. ABE: anonymity benefits; CON: confidence in effectiveness; SKE: skepticism and perception of risk; TET: technologization threat. Note: threshold structure not shown.

In model 1, we examined a 2-factor, hierarchical confirmatory measurement model (2 first-order factors loading on 1 second-order global factor). We posited that the set of attitudes endorsed on the APOI would indicate a “positive attitudes towards internet-based treatments” latent factor as well as a “negative attitudes towards internet-based treatments” latent factor. Indicators drawn from the confidence in effectiveness (CON) and anonymity benefits (ABE) subscales comprise positive attitudes toward iCBT and were tested to examine statistically significant loading onto the “positive” latent factor. Indicators derived from the skepticism and perception of risk (SKE) and technologization threat (TET) subscales of the APOI comprise negative attitudes and were tested for statistically significant loading onto the “negative” latent factor. Both “positive” and “negative” first-order factors loaded onto the second-order global factor (termed *Acceptability* for the purposes of this study; Figure 1).

**Figure 2:**
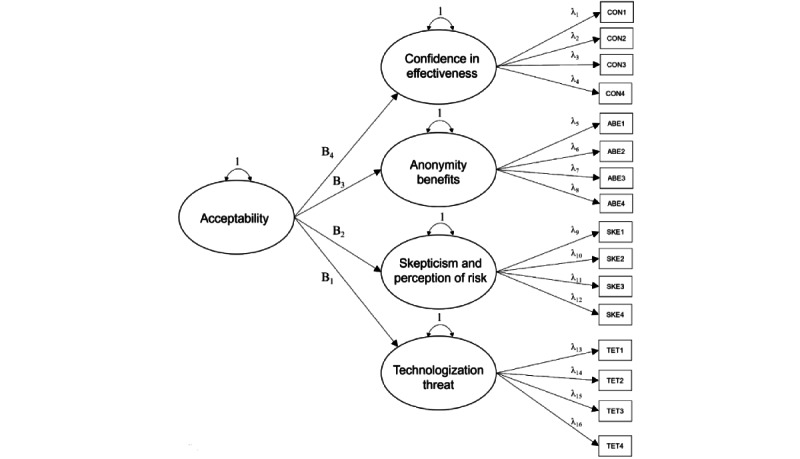
Higher-order, 4-factor model depicting hierarchical relationship among indicators of 4 latent factors: confidence, anonymity benefits, skepticism, and technologization threat loading on a global acceptability factor. ABE: anonymity benefits; CON: confidence in effectiveness; SKE: skepticism and perception of risk; TET: technologization threat. Note: threshold structure not shown.

In model 2, we attempted a replication of the 4-factor, hierarchical confirmatory measurement model (4 first-order factors loading on 1 second-order global factor) proposed in the study by Schröder et al [24]. Indicators drawn from the 4 subscales were modeled per the provided confirmatory factor analysis specifications [24]. All 4 first-order factors (CON, ABE, SKE, and TET) were loaded onto the second-order global factor acceptability (Figure 2).

If neither hypothesized model 1 nor model 2 demonstrates adequate model fit, the modification fit indexes provided by the WLSMV estimation will be reviewed, and the comparative fit of a third alternative model (model 3) will be examined.

**Table 3:** Attitudes Towards Psychological Online Interventions Questionnaire: subscale and item descriptions^a^.

Measure name and scale or item label		Description
**Confidence in effectiveness subscale^b^**		Measures positive attitudes concerning the efficacy and credibility of therapist-assisted iCBT^c^
	CON1	A therapist-assisted iCBT program can help me to recognize the issues that I have to challenge.
	CON2	I have the feeling that a therapist-assisted iCBT can help me.
	CON3	A therapist-assisted iCBT program can inspire me to better approach my problems.
	CON4	I believe that the concept of therapist-assisted iCBT programs makes sense.
**Anonymity benefits subscale^b^**		Measures positive attitudes related to the privacy and confidentiality of using a therapist-assisted iCBT
	ABE1	A therapist-assisted iCBT program is more confidential and discreet than visiting a therapist.
	ABE2	By using a therapist-assisted iCBT program, I can reveal my feelings more easily than with a therapist.
	ABE3	I would be more likely to tell my friends that I use a therapist-assisted iCBT program than that I visit a therapist.
	ABE4	By using a therapist-assisted iCBT program, I do not have to fear that someone will find out that I have psychological problems.
**Skepticism and perception of risk subscale^d^**		Measures negative attitudes concerning the efficacy and security of a therapist-assisted iCBT
	SKE1	Using therapist-assisted iCBT programs, I do not expect long-term effectiveness.
	SKE2	Using therapist-assisted iCBT programs, I do not receive professional support.
	SKE3	It is difficult to implement the suggestions of a therapist-assisted iCBT effectively in everyday life.
	SKE4	Therapist-assisted iCBT programs could increase isolation and loneliness.
**Technologization threat subscale^d^**		Measures negative attitudes related to the independent and remote nature of therapist-assisted iCBT
	TET1	In crisis situations, a therapist can help me better than a therapist-assisted iCBT program.
	TET2	I learn skills to better manage my everyday life from a therapist rather than from a therapist-assisted iCBT program.
	TET3	I am more likely to stay motivated with a therapist than when using a therapist-assisted iCBT program.
	TET4	I do not understand therapeutic concepts as well with a therapist-assisted iCBT.

^a^Response scale (1=totally disagree to 5=totally agree).

^b^Higher scores represent greater acceptability.

^c^iCBT: internet-based cognitive behavioral therapy.

^d^Higher scores indicate lower acceptability.

**Table 4:** Bivariate correlations between the 16 Attitudes Towards Psychological Online Interventions items.

Variable	1	2	3	4	5	6	7	8	9	10	11	12	13	14	15	16
CON^a^1	1	—^b^	—	—	—	—	—	—	—	—	—	—	—	—	—	—
CON2	0.74	1	—	—	—	—	—	—	—	—	—	—	—	—	—	—
CON3	0.76	0.79	1	—	—	—	—	—	—	—	—	—	—	—	—	—
CON4	0.71	0.65	0.75	1	—	—	—	—	—	—	—	—	—	—	—	—
ABE^c^1	0.38	0.46	0.47	0.41	1	—	—	—	—	—	—	—	—	—	—	—
ABE2	0.37	0.42	0.43	0.44	0.72	1	—	—	—	—	—	—	—	—	—	—
ABE3	0.20	0.34	0.26	0.25	0.53	0.56	1	—	—	—	—	—	—	—	—	—
ABE4	0.38	0.41	0.40	0.45	0.61	0.58	0.66	1	—	—	—	—	—	—	—	—
SKE^d^1	−0.05	−0.10	−0.07	0.01	−0.27	−0.31	−0.15	−0.17	1	—	—	—	—	—	—	—
SKE2	−0.01	−0.10	−0.02	0.02	−0.12	−0.30	−0.19	−0.18	0.63	1	—	—	—	—	—	—
SKE3	−0.15	−0.21	−0.15	0.03	−0.19	−0.26	−0.22	−0.15	0.71	0.72	1	—	—	—	—	—
SKE4	−0.09	−0.18	−0.07	0.04	−0.22	−0.28	−0.28	−0.25	0.63	0.69	0.75	1	—	—	—	—
TET^e^1	−0.44	−0.42	−0.50	0.58	−0.42	−0.41	−0.28	−0.33	0.24	0.21	0.24	0.22	1	—	—	—
TET2	−0.36	−0.39	−0.42	0.33	−0.43	−0.45	−0.39	−0.43	0.41	0.34	0.41	0.45	0.63	1	—	—
TET3	−0.39	−0.34	−0.41	0.36	−0.47	−0.38	−0.34	−0.41	0.38	0.25	0.30	0.38	0.66	0.72	1	—
TET4	−0.22	−0.22	−0.29	0.18	−0.45	−0.50	−0.33	−0.40	0.54	0.41	0.48	0.51	0.39	0.68	0.62	1

^a^CON: confidence in effectiveness.

^b^Not applicable.

^c^ABE: anonymity benefits.

^d^SKE: skepticism and perception of risk.

^e^TET: technologization threat.

**Table 5:** Descriptive statistics of the 16 Attitudes Towards Psychological Online Interventions items.

	CON^a^1	CON2	CON3	CON4	ABE^b^1	ABE2	ABE3	ABE4	SKE^c^1	SKE2	SKE3	SKE4	TET^d^1	TET2	TET3	TET4
Values, mean (SD)	3.6 (1.0)	3.4 (1.0)	3.6 (1.0)	3.7 (1.0)	3.3 (1.0)	3.2 (0.09)	3.0 (1.0)	3.2 (1.1)	3.1 (1.2)	3.3 (1.1)	3.1 (1.1)	3.2 (1.1)	2.5 (1.0)	2.7 (1.0)	2.6 (1.0)	2.9 (1.1)
Skew	−0.41	−0.15	−0.51	−0.50	−0.03	0.04	0.01	−0.08	−0.09	−0.19	−0.07	−0.13	0.26	0.03	0.18	0.11
Kurt	0.07	0.24	0.34	0.16	−0.02	0.09	−0.12	−0.14	−0.50	−0.34	−0.18	−0.33	0.30	0.16	0.07	−0.06

^a^CON: confidence in effectiveness.

^b^ABE: anonymity benefits.

^c^SKE: skepticism and perception of risk.

^d^TET: technologization threat.

### Ethics Approval

This study was conducted in compliance with The Georgia State University institutional review board protocol #H18341 and preregistered with the Open Science Framework [45].

## Results

### Sample Characteristics

A total of 268 participants were enrolled in the study and completed the survey. Of these, 14 participants were excluded because they did not complete the APOI questionnaire, thus yielding a sample of 254 participants. Participant ratings suggested mild symptoms of anxiety (mean 8.35, SD 7.10) and stress (mean 11.96, SD 7.88) and normal levels of depressive symptoms (mean 9.00, SD 8.49) according to standard thresholds of the DASS-21 [39].

### Construct Validity

The 2 proposed models explored the construct of acceptability as a hierarchical, 2-factor model comprising “positive attitudes” and “negative attitudes” toward therapist-assisted iCBT, or as a hierarchical, 4-factor model comprising 4 distinct domains of attitudes toward therapist-assisted iCBT (confidence in effectiveness, anonymity benefits, skepticism and perception of risk, and technologization threat). See Table 6 for a full description of the model’s fit indices.

**Figure 3:**
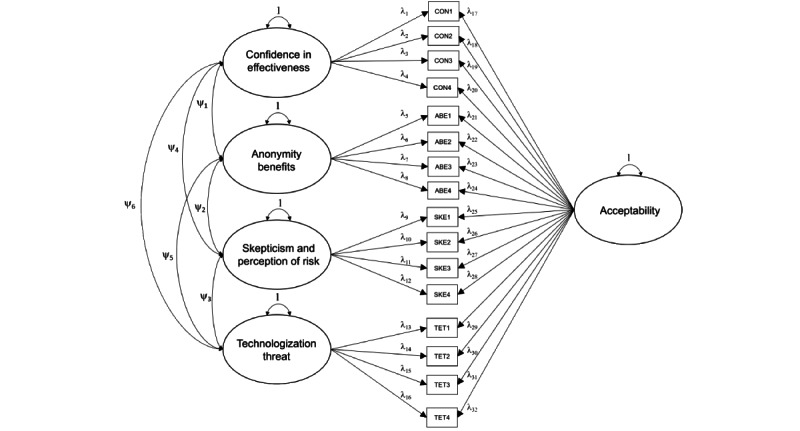
Bifactor model depicting orthogonal relationship among indicators of 4 latent factors: confidence, anonymity benefits, skepticism, and technologization threat loading alongside a global acceptability factor. ABE: anonymity benefits; CON: confidence in effectiveness; SKE: skepticism and perception of risk; TET: technologization threat. Note: threshold structure not shown.

Neither model had a perfect absolute model fit according to the chi-square test (model 1: χ^2^_103_=1579., *P*<.001; model 2: χ^2^_101_=595.3, *P*<.001). There was variation in the absolute values of correlation residuals, as residuals frequently exceeded 0.10 in model 1 (mean 0.14, SD 0.01), contrary to recommendations for ordered categorical variables [36]. Correlation residuals were largely below 0.10 in model 2 (mean 0.07, SD 0.01). Model 1 indicated poor fit according to CFI (0.65), TLI (0.59), SRMR (0.12), and RMSEA (0.24, 90% CI 0.23-0.25). Model 2 demonstrated better fit estimates with CFI (0.88), TLI (0.86), SRMR (0.08), and marginally improved RMSEA (0.14, 90% CI 0.13-0.15). As neither model 1 nor model 2 demonstrated adequate fit indices, an alternative bifactor model 3 (shown in Table 6) was examined because it retains theoretical similarity to the structure proposed by Schröder et al [24], and hierarchical models (ie, model 2) have more parameter constraints and are nested within less constrained bifactor models (ie, model 3) [46-48]. In model 3, the 4 factors (CON, ABE, SKE, and TET) were specified as orthogonal (instead of hierarchical) to the global factor of acceptability (Figure 3). Chi-square tests did not indicate an absolute model fit: χ^2^_82_=248.7, *P*<.001, although the chi-square:df ratio was 3.03, which is within the recommended range between 2 and 5 [44]. Furthermore, model 3 indicated better estimates with CFI=0.96, TLI=0.94, SRMR=0.03, and RMSEA=0.09, 90% CI 0.08-0.10. Overall, model 3 demonstrated adequate to good fit according to accepted thresholds [42-44] and the absolute values of correlation residuals did not exceed 0.10 (mean 0.03, SD 0.002). Other equivalent models were investigated (informed by statistically significant modification indices and theoretical rationale), but none demonstrated both structural fit and conceptual interpretability or parsimony (see Multimedia Appendix 1 for all tested confirmatory factor analysis models).

**Figure 4:**
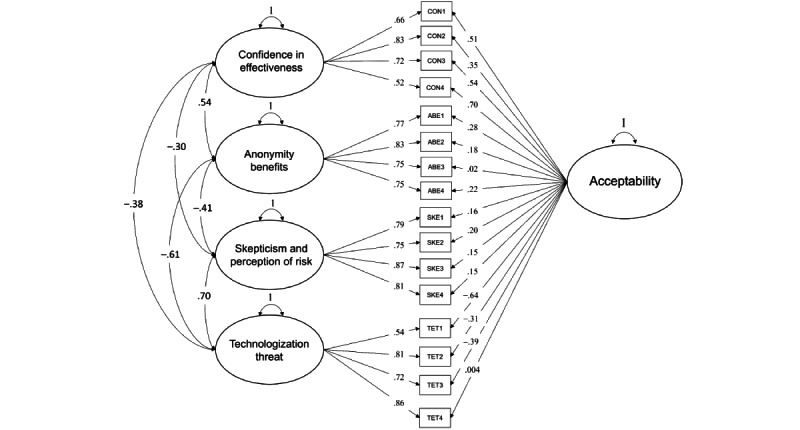
Bifactor model depicting orthogonal relationship among indicators of 4 latent factors: confidence, anonymity benefits, skepticism, and technologization threat loading alongside a global acceptability factor. Standardized parameter estimates shown. ABE: anonymity benefits; CON: confidence in effectiveness; SKE: skepticism and perception of risk; TET: technologization threat. Note: threshold structure not shown.

As models 1, 2, and 3 were nested, comparisons were conducted to verify the statistically improved model fit by examining the change in the chi-square statistic. As the scaled chi-square value for WLSMV cannot be used for traditional chi-square difference testing, the DIFFTEST option in Mplus (version 8.4) was used [49]. As shown in Table 6, comparisons indicated a significant chi-square change, Δχ^2^_2_=327.7, *P*<.001, suggesting that model 2 was significantly better than model 1. Similarly, there was a significant chi-square change, Δχ^2^_19_=231.9, *P*<.001, suggesting that model 3 was significantly better than model 2. Model 3 was the best fitting model and is described in more detail below (see Table 7 for full factor loadings and Figure 4 for the model with parameter estimates).

When examining the standardized factor loadings of the bifactor model, the absolute value of loadings for the categorical indicators ranged from 0.52 to 0.87 on their original 4 factors. Consistent with the findings of Schröder et al [24], all indicators significantly loaded onto their respective latent factors (CON, ABE, SKE, and TET), supporting the theory that these 4 domains are valid indicators of attitudes toward internet-delivered treatment. Furthermore, the 2 positively valenced latent factors (CON and ABE) significantly covaried as similar yet distinct factors (ψ=0.54; *P*<.001) as did the 2 negatively valenced latent factors (SKE, TET; ψ=0.70; *P*<.001).

The relationship between the 16 ordinal indicators and the global acceptability factor was more complex, as the absolute value of the loadings ranged from 0.004 to 0.70. Although the factor loadings for both CON and ABE indicators were positively correlated with the global acceptability factor, only CON indicators demonstrated adequate strength (0.35-0.70), whereas loadings for ABE items ranged from 0.02 to 0.28, suggesting a relatively weak relationship with the global factor. One item of the ABE subscale (ABE3) “I would be more likely to tell my friends that I use a therapist-assisted iCBT program than that I visit a therapist” did not load significantly on the global factor (λ=0.016; *P*=.83). Furthermore, there was significant heterogeneity in the factor loadings for both the SKE and TET indicators on the global factor. Despite its conceptualization as “negative attitudes,” factor loadings of indicators of SKE ranged from 0.15 to 0.20 and were *positively* correlated with the global acceptability factor. Conversely, factor loadings of indicators of TET ranged from 0.39 to 0.64 and were negatively correlated with the global acceptability factor. One item of the TET subscale (TET4) “I do not understand therapeutic concepts as well with a therapist-assisted iCBT as I do with a live therapist” did not load significantly on the global factor (λ=0.004; *P*=.95).

Overall, the results from the bifactor model structure of the APOI provide evidence that the 4 factors proposed by Schröder et al [24] exhibit an orthogonal relationship with the global factor of acceptability. As expected, positively valenced factors were positively related to one another, negatively valenced factors were positively related to one another, and each item was a significant indicator of the 4 distinct subscales when controlling for the common variance shared by the global factor. The bifactor model shows that most (but not all) of the 16 APOI items are significant indicators of the global factor, although all SKE items were related in the opposite direction.

**Table 6:** Goodness-of-fit indexes of models tested in confirmatory factor analysis.

Model name	Chi-square (*df*)	*P* value	CFI^a^	TLI^b^	SRMR^c^	RMSEA^d^ (95% CI)	Comparison		
							ΔChi-square (*df*)	*P* value	Note
2 factor	1579.8 (103)	<.001	0.65	0.59	0.12	0.24 (0.23-0.25)	—^e^	—	—
4 factor^f^	595.3 (101)	<.001	0.88	0.86	0.08	0.14 (0.13-0.15)	984.45 (2)	<.001	Versus model 1
Bifactor^f^	248.7 (82)	<.001	0.96	0.94	0.03	0.09 (0.08-0.10)	346.57 (19)	<.001	Versus model 2

^a^CFI: comparative fit index.

^b^TLI: Tucker-Lewis index.

^c^SRMR: standardized root mean squared residual.

^d^RMSEA: root mean square error of approximation.

^e^Not available.

^f^DIFFTEST command used for weighted least squares means and variance adjusted estimators to test differences in model fit.

**Table 7:** Model 3 (bifactor) standardized factor loadings with SEs.

Relation or variable				Estimate (SE)		*P* value
**Loadings**						
	**Confidence in effectiveness (CON) BY**					
		CON1	0.66 (0.06)		<.001	
		CON2	0.83 (0.04)		<.001	
		CON3	0.72 (0.06)		<.001	
		CON4	0.52 (0.07)		<.001	
	**Anonymity benefits (ABE) BY**					
		ABE1	0.77 (0.03)		<.001	
		ABE2	0.83 (0.03)		<.001	
		ABE3	0.75 (0.03)		<.001	
		ABE4	0.75 (0.03)		<.001	
	**Skepticism and perception of risk (SKE) BY**					
		SKE1	0.79 (0.02)		<.001	
		SKE2	0.75 (0.03)		<.001	
		SKE3	0.87 (0.02)		<.001	
		SKE4	0.81 (0.02)		<.001	
	**Technologization threat (TET) BY**					
		TET1	0.54 (0.06)		<.001	
		TET2	0.81 (0.03)		<.001	
		TET3	0.72 (0.04)		<.001	
		TET4	0.86 (0.03)		<.001	
	**Acceptability BY**					
		CON1	0.51 (0.07)		<.001	
		CON2	0.35 (0.08)		<.001	
		CON3	0.54 (0.08)		<.001	
		CON4	0.70 (0.07)		<.001	
		ABE1	0.28 (0.07)		<.001	
		ABE2	0.18 (0.08)		.01	
		ABE3	0.02 (0.08)		.83	
		ABE4	0.22 (0.07)		.001	
		SKE1	0.16 (0.06)		.01	
		SKE2	0.20 (0.06)		.001	
		SKE3	0.15 (0.06)		.02	
		SKE4	0.15 (0.06)		.008	
		TET1	−0.64 (0.05)		<.001	
		TET2	−0.31 (0.07)		<.001	
		TET3	−0.39 (0.07)		<.001	
		TET4	<.01 (0.08)		.95	
**Factor covariances**						
	**Confidence in effectiveness WITH**					
		Anonymity benefits	0.54 (0.06)		<.001	
		Skepticism and perception of risks	−0.30 (0.05)		<.001	
		Technologization threat	−0.38 (0.06)		<.001	
		Acceptability	0.00 (—^a^)		—	
	**Anonymity benefits WITH**					
		Skepticism and perception of risks	−0.41 (0.06)		<.001	
		Technologization threat	−0.61 (0.05)		<.001	
		Acceptability	0.00 (—)		—	
	**Skepticism and perception of risk WITH**					
		Technologization threat	0.70 (0.05)		<.001	
		Acceptability	0.00 (—)		—	
	**Technologization threat WITH**					
		Acceptability	0.00 (—)		—	

^a^Not available.

### Reliability

The APOI demonstrated excellent internal consistency for the total scale (Cronbach α=.89) and retained good-to-excellent reliability across subscales (Cronbach α=.84 for ABE, .85 for TET, .87 for SKE, and .90 for CON). Across subscales, the corrected item-total correlations ranged from 0.59 to 0.83, with a mean adjusted correlation of 0.71 indicating good item discrimination within subscales. The corrected item‐total correlations for the APOI total scale ranged from 0.45 to 0.68, with a mean adjusted correlation of 0.55, indicating good item discrimination within the total scale.

## Discussion

### Principal Findings

This study evaluated the psychometric properties of the APOI questionnaire [24], which is the most robust and widely used measure of *acceptability* for digital mental health interventions within a sample of Black Americans. The APOI demonstrated good-to-excellent internal consistency in the current sample, both as a total score and across subscales (Cronbach α=.84−.90), which is stronger than the internal consistency reported in the original publication (Cronbach α=.62−.77).

However, the original hierarchical, 4-factor model proposed by Schröder et al [24] exhibited relatively poor goodness-of-fit indices. Instead, the APOI showed the strongest evidence for construct validity of a bifactor model in which each of the indicators loaded on a global factor of acceptability and the global factor of acceptability was orthogonally related to the 4 subscales. Although this unexpected finding is inconsistent with the hierarchical model proposed by Schröder et al [24], it is consistent with the literature showing that bifactor models fit better than their equivalent higher-order model in more than 90% of comparisons for mental abilities test batteries [50] and can be particularly valuable in evaluating the plausibility of subscales [51,52]. The strong, positive correlations between positively valenced subscales (confidence in effectiveness and anonymity benefits) and negatively valenced subscales (skepticism and perception of risk and technologization threat), and the negative correlations across oppositely valenced subscales are compelling evidence that the subscales have meaningful discriminant validity and can be interpreted in their own right.

The heterogeneity of findings regarding model fit may be explained by the nature of the coefficients of the factor loadings and overall structure. Modeling both positive and negatively valenced factors onto a unitary, higher-order construct (ie, acceptability) can prove difficult, especially when variance exists among indicators of lower-order constructs. The factor loadings between the 16 indicators and global acceptability factor varied substantially. Several indicators loading on the ABE, SKE, and TET subscales exhibited relatively weak or null relations with acceptability or were in the opposite direction than expected. Items loaded on the ABE subscale, in particular, may indicate both facilitators and barriers to engagement with digital interventions, given the user’s conflicting perceptions of digital privacy and confidentiality [8]. Items that loaded on the SKE subscale were positively correlated with acceptability which is contrary to the conceptualization of this subscale as a construct reflecting negative attitudes, although this is interpreted with caution, given their weak correlations.

Scholars have called for better conceptualizations of acceptability [15,23], which have the potential to produce even more parsimonious measures by exploring new factors or consolidating indicators to reduce conceptual overlap. In particular, there is a growing need for evidence of the dimensions of acceptability that are demonstrably correlated with uptake, engagement, and adherence to digital mental health interventions. As discussed in prior research, this apparent discrepancy in consumer attitudes and behaviors may, in fact, be a consequence of the heterogeneous nature and definition of acceptability toward digital mental health interventions [22,24]. A considerable amount of research uses a single item to assess acceptability and results from this study, and others [29,30,32], demonstrate that single-items measures are inadequate for the operationalization of this heterogeneous construct.

Furthermore, these data suggest that within a Black American population, there is greater utility in interpreting the APOI subscales as attitudinal constructs distinct from a global acceptability factor. However, given that the higher-order model is nested within the bifactor model [46-48], these models are not necessarily at odds with one another. Ultimately, these results provide support for the underlying validity of the 4 factors proposed by the APOI but eschew traditional practices of prioritizing the calculation of a single acceptability score at the expense of adequately measuring each relevant dimension of acceptability and reporting them in tandem with the global score for contextualization.

### Strengths and Limitations

This is the first study to investigate the psychometric properties of the APOI questionnaire among a racially minoritized population. This study is the first to provide evidence for the cross-cultural equivalence of APOI among Black Americans. This is a notable contribution to the literature, as the vast majority of randomized controlled trials examining the efficacy and acceptability of iCBT do not include (or do not report) racial minorities in their studies [13], and existing measures of consumer attitudes toward digital mental health interventions [24,29-33] have predominantly been developed and examined for validation within White majority (and predominantly European) samples. Furthermore, by modifying the target treatment from “psychological online interventions” to “therapist-assisted iCBT,” this study provides preliminary evidence for the utility of the APOI for diverse digital interventions with varying degrees of specificity. Overall, the results suggest that the APOI is a robust measure.

Despite the strengths of this study, there are some limitations that warrant attention. The study sample consisted of participants with minimal symptoms of depression, anxiety, or stress. This was distinct from the participants who reported moderate levels of depression in the study by Schröder et al [24]. Future research needs to evaluate these measures among those with greater depression severity or other diagnoses. The participants in this study were predominantly young adult females. These demographic groups are more likely to use digital mental health interventions, and the relative impact of their positive and negative attitudes towards digital mental health intervention is likely to differ across diverse populations [8]. Relatedly, measurement invariance was not formally assessed across different subgroups within the sample (eg, male vs female), because of significant imbalances in sample size, which minimized the power to detect potential differences between these groups. Finally, the convergent validity of the APOI with other measures of acceptability within a Black American sample could not be determined because no other relevant measures of acceptability existed at the time of data collection for this study.

### Future Directions

Future research should modify the APOI to apply it to other digital mental health interventions (eg, virtual reality exposure therapies and massively open web-based interventions) and translate the measure into additional languages (eg, Spanish) to further examine cross-intervention and cross-cultural equivalency. Although the APOI demonstrated good internal consistency reliability within the present sample, test-retest reliability was not examined. Indeed, with the exception of the study by Clough et al [29], there is a notable lack of investigation of the test-retest reliability of acceptability measures, which deserves further evaluation. Moreover, it would be compelling to investigate the criterion validity of the APOI to examine whether positive attitudes toward digital mental health interventions predict the willingness to use or actual use of digital mental health interventions among racially and ethnically minoritized participants. Consistent with the Theory of Planned Behavior [53], which emphasizes the relationship among beliefs, attitudes, and behavioral intentions, positive attitudes toward acceptability would be expected to be the strongest predictor of behavioral intention, which in turn is the immediate determinant of actual treatment-seeking behavior. Investigations of the relationship between attitudes toward iCBT and the effectiveness of such interventions should be conducted, as those with more positive attitudes might derive greater clinical benefits. Finally, although studies examining the convergent validity of the APOI with related measures of acceptability toward digital mental health interventions have been recently conducted [29,30], these studies did not expressly recruit participants from racially and ethnically minoritized communities, and their results are predominantly based on White or European samples. This is concerning, as racially and ethnically minoritized communities may be positioned to benefit the most from the treatment accessibility advantages afforded by digital mental health interventions [54]. Understanding these communities’ attitudes toward these treatments is paramount.

### Conclusions

The APOI questionnaire is a valid and reliable measure of attitudes toward therapist-assisted iCBT among Black Americans. However, some of the indicators were only weakly associated with the global factor of acceptability, and a bifactor model demonstrated better goodness-of-fit than the hierarchical, 4-factor structure proposed by the original authors. This provides strong evidence that the APOI demonstrates multidimensionality and that there is greater utility in interpreting APOI subscales as attitudinal constructs distinct from a global acceptability factor. Indeed, attitudes of acceptability comprise both positive and negative attitudes toward the uptake of digital mental health interventions and must be evaluated in tandem to effectively understand the nuanced attitudes consumers may hold toward these interventions. This is the first study to examine the psychometric properties of any measure of consumer attitudes toward digital mental health interventions among Black participants. Demonstrating the reliability, validity, and cultural equivalency of existing measures of attitudes toward these interventions is needed to improve our understanding of the drivers of and barriers to using digital treatments among minoritized communities. For the full potential of digital mental health interventions to improve equitable access to treatment to be realized, more adequate representation of minoritized communities in research on these interventions must be achieved.

## Appendix

Multimedia Appendix 1 Materials depict fit indices for all examined confirmatory factor analyses. Mplus (version 8.4) syntax is provided for all analyses.

## Abbreviations

APOI: Attitudes Towards Psychological Online Interventions
CFI: comparative fit index
DASS-21: Depression Anxiety Stress Scale-21 items
iCBT: internet-based cognitive behavioral therapy
RMSEA: root mean square error of approximation
SRMR: standardized root mean squared residual
TLI: Tucker-Lewis index
WLSMV: weighted least squares means and variance adjusted
