# Supplementary material for: Validation of the Attitudes Towards Psychological Online Interventions Questionnaire Among Black Americans: Cross-cultural Confirmatory Factor Analysis
Source: JMIR Ment Health. 2023 Apr 27;10:e43929. doi: 10.2196/43929 (PMC10176146; doi:10.2196/43929)
Supplement: Multimedia Appendix 1 [file mental_v10i1e43929_app1.docx]

## Supplemental Materials

### S1: Fit Indices of A Priori and Exploratory CFA Models

|  | | |  |  |  |  |  |
| --- | --- | --- | --- | --- | --- | --- | --- |
| Model Name | χ ^2^ | *df* | *p* | CFI | TLI | SRMR | RMSEA [90% CI] |
| 1. 2^nd^ Order Two Factor | 1579.76 | 103 | <.01 | .65 | .59 | .12 | .24 [.23 -.25] |
| 2. 2^nd^ Order Four Factor | 595.31 | 101 | <.01 | .88 | .86 | .08 | .14 [.13 -.15] |
| 3. Bifactor^a^ | 248.74 | 82 | <.01 | .96 | .94 | .03 | .09 [.08 -.10] |
| 4. One Factor^a^ | 2577.55 | 104 | <.01 | .41 | .32 | .15 | .31 [.30 - .32] |
| 5. Two Factor^a^ | 1579.76 | 103 | <.01 | .65 | .59 | .12 | .24 [.23 -.25] |
| 6. Four Factor^a^ | 505.48 | 98 | <.01 | .90 | .88 | .06 | .13 [.12-.14] |

*Note.* CFA = Confirmatory Factor Analysis; CFI = Comparative Fit Index; TLI = Tucker-Lewis Index; RMSEA = Root Mean Square Error of Approximation, SRMR = Standardized Root Mean Squared Residual
Recommended Cut-offs: CFI ≥ .90; TLI ≥ .90; χ^2^ / *df* ≤ 5; RMSEA ≤ .08; SRMR ≤ .08

^a^Exploratory Models

### S2: Model 1 (Higher-Order, Two-Factor Model: *Mplus 8.4* Syntax)

TITLE:

Second Order Two-Factor CFA: Acceptability of TA-iCBT;

DATA:

FILE IS "C:\Users\dellis22\Desktop\Ellis_CFA\APOI.dat";

VARIABLE:

NAMES ARE

PID CON1 CON2 CON3 CON4 ABE1 ABE2 ABE3 ABE4

SKE1 SKE2 SKE3 SKE4 TET1 TET2 TET3 TET4;

USEVARIABLES ARE

CON1 CON2 CON3 CON4 ABE1 ABE2 ABE3 ABE4

SKE1 SKE2 SKE3 SKE4 TET1 TET2 TET3 TET4;

CATEGORICAL ARE ALL;

MISSING ARE ALL (-99);

ANALYSIS:

ESTIMATOR IS WLSMV;

ITERATIONS = 1000;

CONVERGENCE = 0.00001;

MODEL:

! first order free the first indicator;

Positive BY CON1* CON2 CON3 CON4 ABE1 ABE2 ABE3 ABE4;

Negative BY SKE1* SKE2 SKE3 SKE4 TET1 TET2 TET3 TET4;

! second order;

Accept BY Positive Negative;

! fix the latent variance;

Positive@1 Negative@1 Accept@1;

! suppress 1^st^ order covariances;

Positive WITH Negative@0;

! variances for categorical variables not estimated;

! threshold structure is modeled by default;

OUTPUT: sampstat modindices(all,0)residual STDYX ; !standardized;STDYX;

### S3: Model 2 (Higher-Order, Four-Factor Model: *Mplus 8.4* Syntax)

TITLE:

Second Order- Four Factor CFA: Acceptability of TA-iCBT;

DATA:

FILE IS "C:\Users\dellis22\Desktop\Ellis_CFA\APOI.dat";

VARIABLE:

NAMES ARE

PID CON1 CON2 CON3 CON4 ABE1 ABE2 ABE3 ABE4

SKE1 SKE2 SKE3 SKE4 TET1 TET2 TET3 TET4;

USEVARIABLES ARE

CON1 CON2 CON3 CON4 ABE1 ABE2 ABE3 ABE4

SKE1 SKE2 SKE3 SKE4 TET1 TET2 TET3 TET4;

CATEGORICAL ARE ALL;

MISSING ARE ALL (-99);

ANALYSIS:

ESTIMATOR IS WLSMV;

ITERATIONS = 1000;

CONVERGENCE = 0.00001;

MODEL:

! first order free the first indicator;

Confidence BY CON1* CON2 CON3 CON4;

Anonymous BY ABE1* ABE2 ABE3 ABE4;

Skeptic BY SKE1* SKE2 SKE3 SKE4;

Technology BY TET1* TET2 TET3 TET4;

! second order;

Accept BY Confidence Anonymous Skeptic Technology;

! fix the latent variance;

Confidence@1 Anonymous@1 Skeptic@1 Technology@1 Accept@1;

! suppress 1^st^ order covariances;

Confidence WITH Anonymous@0 Skeptic@0 Technology@0;

Anonymous WITH Skeptic@0 Technology@0;

Skeptic WITH Technology@0;

SAVEDATA:

Difftest is mydiff1.dat;

! variances for categorical variables not estimated;

! threshold structure is modeled by default;

OUTPUT: sampstat modindices(all,0) residual STDYX ; !standardized;STDYX;

### S4: Model 3 (Bifactor, Global Model: *Mplus 8.4* Syntax)

TITLE:

Bifactor - Global - CFA: Acceptability of TA-iCBT;

DATA:

FILE IS "C:\Users\dellis22\Desktop\Ellis_CFA\APOI.dat";

VARIABLE:

NAMES ARE

PID CON1 CON2 CON3 CON4 ABE1 ABE2 ABE3 ABE4

SKE1 SKE2 SKE3 SKE4 TET1 TET2 TET3 TET4;

USEVARIABLES ARE

CON1 CON2 CON3 CON4 ABE1 ABE2 ABE3 ABE4

SKE1 SKE2 SKE3 SKE4 TET1 TET2 TET3 TET4;

CATEGORICAL ARE ALL;

MISSING ARE ALL (-99);

ANALYSIS:

ESTIMATOR IS WLSMV;

ITERATIONS = 1000;

CONVERGENCE = 0.00001;

MODEL:

! first order free the first indicator;

Confidence BY CON1* CON2 CON3 CON4;

Anonymous BY ABE1* ABE2 ABE3 ABE4;

Skeptic BY SKE1* SKE2 SKE3 SKE4;

Technology BY TET1* TET2 TET3 TET4;

Accept BY CON1* CON2 CON3 CON4 ABE1 ABE2 ABE3 ABE4

SKE1 SKE2 SKE3 SKE4 TET1 TET2 TET3 TET4;

! fix the latent variance;

Confidence@1 Anonymous@1 Skeptic@1 Technology@1 Accept@1;

! suppress covariances between bifactor groups;

Confidence WITH Anonymous Skeptic Technology Accept@0;

Anonymous WITH Skeptic Technology Accept@0;

Skeptic WITH Technology Accept@0;

Technology WITH Accept@0;

SAVEDATA:

Difftest is mydiff2.dat;

! variances for categorical variables not estimated;

! threshold structure is modeled by default;

OUTPUT: sampstat modindices(all,0) residual STDYX; !standardized;STDYX;

### S5: Model 4 (One-Factor Model: *Mplus 8.4* Syntax)

TITLE:

One Factor CFA: Acceptability of TA-iCBT;

DATA:

FILE IS "C:\Users\dellis22\Desktop\Ellis_CFA\APOI.dat";

VARIABLE:

NAMES ARE

PID CON1 CON2 CON3 CON4 ABE1 ABE2 ABE3 ABE4

SKE1 SKE2 SKE3 SKE4 TET1 TET2 TET3 TET4;

USEVARIABLES ARE

CON1 CON2 CON3 CON4 ABE1 ABE2 ABE3 ABE4

SKE1 SKE2 SKE3 SKE4 TET1 TET2 TET3 TET4;

CATEGORICAL ARE ALL;

MISSING ARE ALL (-99);

ANALYSIS:

ESTIMATOR IS WLSMV;

ITERATIONS = 1000;

CONVERGENCE = 0.00001;

MODEL:

! first order free the first indicator;

Accept BY CON1* CON2 CON3 CON4 ABE1 ABE2 ABE3 ABE4

SKE1 SKE2 SKE3 SKE4 TET1 TET2 TET3 TET4;

! fix the latent variance;

Accept@1;

! variances for categorical variables not estimated;

! threshold structure is modeled by default;

OUTPUT: sampstat modindices(all,0) residual STDYX; !standardized;STDYX;

### S6: Model 5 (Two-Factor Model: *Mplus 8.4* Syntax)

TITLE:

Two-Factor CFA: Acceptability of TA-iCBT;

DATA:

FILE IS "C:\Users\dellis22\Desktop\Ellis_CFA\APOI.dat";

VARIABLE:

NAMES ARE

PID CON1 CON2 CON3 CON4 ABE1 ABE2 ABE3 ABE4

SKE1 SKE2 SKE3 SKE4 TET1 TET2 TET3 TET4;

USEVARIABLES ARE

CON1 CON2 CON3 CON4 ABE1 ABE2 ABE3 ABE4

SKE1 SKE2 SKE3 SKE4 TET1 TET2 TET3 TET4;

CATEGORICAL ARE ALL;

MISSING ARE ALL (-99);

ANALYSIS:

ESTIMATOR IS WLSMV;

ITERATIONS = 1000;

CONVERGENCE = 0.00001;

MODEL:

! first order free the first indicator;

Positive BY CON1* CON2 CON3 CON4 ABE1 ABE2 ABE3 ABE4;

Negative BY SKE1* SKE2 SKE3 SKE4 TET1 TET2 TET3 TET4;

! fix the latent variance;

Positive@1 Negative@1;

SAVEDATA:

Difftest is mydiff2.dat;

! variances for categorical variables not estimated;

! threshold structure is modeled by default;

OUTPUT: sampstat modindices(all,0)residual STDYX ; !standardized;STDYX;

### S7: Model 6 (Four-Factor Model: *Mplus 8.4* Syntax)

TITLE:

Four-Factor CFA: Acceptability of TA-iCBT;

DATA:

FILE IS "C:\Users\dellis22\Desktop\Ellis_CFA\APOI.dat";

VARIABLE:

NAMES ARE

PID CON1 CON2 CON3 CON4 ABE1 ABE2 ABE3 ABE4

SKE1 SKE2 SKE3 SKE4 TET1 TET2 TET3 TET4;

USEVARIABLES ARE

CON1 CON2 CON3 CON4 ABE1 ABE2 ABE3 ABE4

SKE1 SKE2 SKE3 SKE4 TET1 TET2 TET3 TET4;

CATEGORICAL ARE ALL;

MISSING ARE ALL (-99);

ANALYSIS:

ESTIMATOR IS WLSMV;

ITERATIONS = 1000;

CONVERGENCE = 0.00001;

MODEL:

! first order free the first indicator;

Confidence BY CON1* CON2 CON3 CON4;

Anonymous BY ABE1* ABE2 ABE3 ABE4;

Skeptic BY SKE1* SKE2 SKE3 SKE4;

Technology BY TET1* TET2 TET3 TET4;

! fix the latent variance;

Confidence@1 Anonymous@1 Skeptic@1 Technology@1;

SAVEDATA:

Difftest is mydiff2.dat;

! variances for categorical variables not estimated;

! threshold structure is modeled by default;

OUTPUT: sampstat modindices(all,0) residual STDYX; !standardized;STDYX;
